# Lions in a coexistence landscape: Repurposing a traditional field technique to monitor an elusive carnivore

**DOI:** 10.1002/ece3.8662

**Published:** 2022-03-01

**Authors:** Guy Western, Nicholas B. Elliot, Steiner L. Sompeta, Femke Broekhuis, Shadrack Ngene, Arjun M. Gopalaswamy

**Affiliations:** ^1^ South Rift Association of Landowners Nairobi Kenya; ^2^ 98955 Kenya Wildlife Trust Nairobi Kenya; ^3^ Department of Zoology Wildlife Conservation Research Unit Recanati‐Kaplan Centre University of Oxford Oxford UK; ^4^ Wildlife Ecology and Conservation Group Wageningen University and Research Wageningen The Netherlands; ^5^ Wildlife Research and Training Institute Naivasha Kenya; ^6^ 2015 Carnassials Global Bengaluru India; ^7^ Wildlife Conservation Society Global Conservation Programs Bronx New York USA

**Keywords:** abundance, African lion, coexistence, Maasailand, noninvasive sampling, spatially‐explicit capture‐recapture

## Abstract

Throughout Africa, lions are thought to have experienced dramatic population decline and range contraction. The greatest declines are likely occurring in human‐dominated landscapes where reliably estimating lion populations is particularly challenging. By adapting a method that has thus far only been applied to animals that are habituated to vehicles, we estimate lion density in two community areas in Kenya's South Rift, located more than 100 km from the nearest protected area (PA). More specifically, we conducted an 89‐day survey using unstructured spatial sampling coupled with playbacks, a commonly used field technique, and estimated lion density using spatial capture‐recapture (SCR) models. Our estimated density of 5.9 lions over the age of 1 year per 100 km^2^ compares favorably with many PAs and suggests that this is a key lion population that could be crucial for connectivity across the wider landscape. We discuss the possible mechanisms supporting this density and demonstrate how rigorous field methods combined with robust analyses can produce reliable population estimates within human‐dominated landscapes.

## Graphical abstract

African lions are thought to be declining rapidly, and particularly in human‐dominated landscapes, where they are most difficult to reliably monitor. We repurposed a traditional field technique, playbacks, and demonstrate how to incorporate the resulting data into a SCR framework to provide robust density estimates.

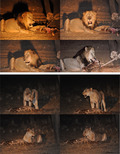

## 1 INTRODUCTION

Recent estimates suggest that lions (*Panthera leo*) have undergone a 43% population decline between 1993 and 2014 (Bauer et al., 2016) and that remaining lion populations in West, Central, and East Africa may decline by 50% over the next two decades (Bauer et al., 2015). Furthermore, lions may occupy as little as 8% of their historic range (Bauer et al., 2016). Such estimates are plagued with uncertainty due to the difficulties of estimating abundance and distribution of these cryptic and wide‐ranging species, difficulties that are amplified in human‐dominated landscapes (Funston & Henschel, 2018). However, it is within these landscapes that the greatest numeric and geographic declines are likely to have occurred resulting in calls to focus conservation efforts in and around designated protected areas (PAs) by bolstering the levels of funding and protection (Lindsey et al., 2017, 2018). While PAs are frequently perceived to be bastions of hope for lion conservation, they may only encompass ~56% of current lion range (Lindsey et al., 2018), and lions and their prey may also be declining in a high proportion of Africa's PAs (Lindsey et al., 2017). The remaining ~44% of lion range falls within a mosaic of land uses from human‐dominated landscapes to private land and conservancies. In many cases, land use is mixed, with people, livestock, and lions sharing the same space. Where tourism is not the primary activity, lions are typically unhabituated to vehicles, illusive, and cryptic making them harder to monitor with direct methods, such as those that involve close‐up photography for individual identification. However, since such areas host a significant proportion of lion range, there is an urgent need for robust density estimation methods.

Over the last decade, practitioners interested in estimating wildlife densities have developed, adapted, and applied spatial capture‐recapture (SCR) models to fit a wide variety of data types and collection protocols (Royle et al., 2013). This flexibility, coupled with a sound theoretical and statistical framework, has seen SCR methods rapidly emerge as the preferred option for many large carnivore population monitoring programs (e.g. Bischof, Milleret, et al., 2020; Duangchantrasiri et al., 2016). A key data requirement is the unambiguous individual identity of detected animals (but see Augustine et al., 2018; Chandler & Royle, 2013; Dey, Delambady, Karanth, et al., 2019 for analytical options involving unidentifiable and partially identifiable individuals). To identify individual carnivores within an SCR framework, researchers generally rely on DNA or unique pelage markings (see Gopalaswamy, Royle, Delampady, et al., 2012 for an example combining these two data sources). To obtain DNA, a variety of hair traps have been used (e.g. Boulanger et al., 2018; Roffler et al., 2019), while scat sampling is also common (e.g. López‐Bao et al., 2018), and may be particularly useful at large spatial scales (e.g. Bischof, Milleret, et al., 2020). For species with unique coat patterns, camera traps are frequently the tool of choice (for a review see Green et al., 2020). While SCR models were originally designed for structured data, Russell et al. (2012) extended these to accommodate unstructured search effort as they conducted foot‐based searches for mountain lions (*Puma concolor*), which were then treed by trained dogs and biopsy darted.

Similar studies have been conducted on African lions and cheetahs (*Acinonyx jubatus*), combining unstructured vehicle‐based searches during daylight with close‐up photography (Braczkowski et al., 2020; Broekhuis et al., 2021; Elliot et al., 2020; Elliot & Gopalaswamy, 2017). However, these studies occurred in areas where lions were habituated to vehicles and relatively easy to find, photograph, and individually identify, potentially limiting the applicability of these techniques to PAs. Here we adapt the approach taken by Elliot and Gopalaswamy (2017) to estimate lion densities in an unprotected area by repurposing a traditional field method, playbacks, and combining this with unstructured search encounter protocols, conducted primarily at night. Despite taking most ID photographs at night, we did not use any night vision equipment, and all photographs were taken using a spotlight and relatively inexpensive DSLR cameras.

## 2 METHODS

### 2.1 Study area

Almost 100 km from the nearest PA (Maasai Mara), this survey was conducted in Shompole and Olkiramatian, two communally owned and managed areas in Kenya's South Rift Ecosystem (Figure A1). The ~358 km^2^ study area (1°58 S, 36° 21 E) is semi‐arid, with 400–600 mm mean annual rainfall (Russell et al., 2018). High evapotranspiration rates and low rainfall result in little standing water outside the rainy season. The perennial Ewaso Ngiro River bisects the area—east of the river is designated for permanent settlement and used for wet season livestock grazing; west of the river is used for dry season livestock grazing and also acts as a wildlife conservancy (Figure A1). Livestock rearing occurs in both communities and crop farming occurs to the north‐west of Olkiramatian and the south‐west of Shompole (Russell et al., 2018). The most abundant prey species for lions within the survey area were wildebeest (*Connochaetes taurinus*, ~3.3/km^2^) and zebra (*Equus quagga*, ~6.7/km^2^), with cattle (~6.2/km^2^) and sheep and goats (~52.2/km^2^) being the primary domestic stock (Russell et al., 2018).

### 2.2 Field methods

Fieldwork was conducted over an 89‐day period (September 17, 2018 to December 14, 2018) using two vehicle‐based protocols to locate lions. The search encounter protocol consisted of an observation team covering the study area in a uniform manner while actively searching for lions and fresh tracks and recording their search effort (see Elliot & Gopalaswamy, 2017). When sampling at night, a powerful spotlight was used to scan the surroundings from an elevated position as the vehicle moved. The playback protocol entailed broadcasting sounds at 95DB to attract lions at night. Playback sites were chosen either opportunistically or when fresh tracks had been found. On all occasions, sections of an opportunistically collected carcass of either a giraffe (*Giraffa camelopardalis*) or a plains zebra were used as a bait. Upon arrival at a site, observers tied the bait to a tree, positioned the vehicle in a hidden area that allowed for photographing the baited area without moving and waited in silence for 10 min, then broadcast for 5 min, repeating this cycle, and rotating the speaker 90° until four broadcasts had been completed or lions had appeared. Each playback lasted a maximum of 70 min. We used standard sounds that have been successfully used to attract lions in traditional playback surveys (buffalo [*Syncerus caffer*] calf in distress, squealing pig, squabbling hyaenas [*Crocuta crocuta*], or if male lions were thought to be in the area we used a recording of a lone sub‐adult male lion roaring; e.g., Cozzi et al., 2013). The spatial location and date of each playback were recorded.

When lions were located, a series of close‐up photographs were taken of each lion whenever possible. All photographs were taken with DSLR cameras (Nikon D90 with a 300 mm F4 lens). When lions were sighted at night, a white‐light spotlight was switched on before slowly focusing it on the animal and taking photographs with the aid of the inbuilt camera flash. The photographs were later used to unambiguously identify individuals based on their unique whisker vibrissae spots and other distinguishing features (Pennycuick & Rudnai, 1970), exclude individuals under the age of one year based on phenotypic features (Miller et al., 2016), assign gender based on secondary sexual characteristics, and finally build capture histories (for details see Elliot et al., 2020).

### 2.3 Analytical framework

We used Bayesian SCR models to estimate key state variables. To model the spatial distribution of lions (state process), we first generated a state‐space by adding a 15 km buffer around the sampled area (Royle et al., 2013). Next, we generated equally spaced pixels (0.5 km^2^) representing potential activity centers across the 2358 km^2^ state‐space and masked out agricultural areas and large water bodies as unsuitable habitat (Figure A1). The value of *M* (the data‐augmented value of abundance in the larger state‐space) was set at 250 (Royle et al., 2009).

To describe the manner in which individuals were detected during the survey (observation process), we compiled a standard SCR array (Gopalaswamy, Royle, Hines, et al., 2012) consisting of individuals, trap locations (defined by grid cells of 1 km^2^), and sampling occasions. Here a trap refers to any grid cell within which we invested either search encounter or playback effort during the survey. Since the intensity and type of effort invested might influence detection rates, we included trap and sampling occasion‐specific covariates for the search encounter protocol and the playback protocol. Including these detection covariates extends the SCR model used in Elliot and Gopalaswamy (2017) to accommodate multiple field protocols that can be adapted to local field conditions. We included sex‐specific covariates when defining the observation process.

### 2.4 Candidate models

We defined four a priori models that assumed (1) both the basal encounter rate $\left(\lambda_{0}\right)$ and the rate of decline in detection probability $\left(\sigma \right)$ are sex‐specific, (2) $\lambda_{0}$ is not sex‐specific but σ is sex‐specific, (3) neither $\lambda_{0}$ nor σ is sex‐specific and (4) $\lambda_{0}$ is sex‐specific but σ is not sex‐specific (see Table 1 for parameter definitions). Each candidate model included both the search encounter protocol (effort), taken in the logarithmic scale, and the playback protocol (effort_2_), taken as an indicator (0 or 1) variable. The detection function parameter $\left(\theta \right)$ was fixed to 1, which implies a fixed half‐normal detection function. The probability of detecting lion *i* in pixel *j* on sampling occasion *k* $\left(\pi_{\mathrm{ijk}}\right)$ is defined by a complementary log‐log function of covariates:

$$\text{cloglog}\left(\pi_{\mathrm{ijk}}\right)=\text{log}\lambda_{0}+\beta_{\text{eff}}\left[\log\left({\text{effort}}_{\mathrm{jk}}\right)\right]+\beta_{\text{eff}2}\left[{\text{effort}}_{2jk}\right]+\beta_{\text{sex}}\left({\text{sex}}_{i}\right)‐f\left[\text{dist}\left(i,j\right)|\theta ,\sigma_{\text{sex}}\right]$$

where $f\left[\text{dist}\left(i,j\right)|\theta ,\sigma_{\text{sex}}\right]$ describes how detection rate is a function of distance between the activity center of individual i and pixel j, which are conditional on θ and $\sigma_{\text{sex}}$.

**TABLE 1:** Posterior summaries of parameters estimated from a Bayesian spatial capture‐recapture model used to estimate spatial lion density in Shompole and Olkiramatian community areas in Kenya

Parameter	Definition	Posterior mean	Posterior standard deviation	95% Lower HPD	95% Upper HPD
$\sigma_{F}$	Rate of decline in detection probability as a female lion's activity center increases as a function of her distance from the centroid of a sampled grid cell	1.82	0.21	1.42	2.23
$\sigma_{M}$	Rate of decline in detection probability as a male lion's activity center increases as a function of his distance from the centroid of a sampled grid cell	2.00	0.37	1.36	2.75
$\beta_{\text{sex}}$	Difference of the complementary log‐log value of detection probability between a male and a female lion	−0.33	0.45	−1.21	0.55
$\beta_{\text{eff}}$	The rate of change in the complementary log‐log value of detection probability as the (log) effort changes by one unit, where effort is measured in kilometers	3.65	0.25	3.15	4.14
$\beta_{\text{eff}2}$	The change in the complementary log‐log value of detection probability when playbacks are included	0.28	0.83	−1.46	1.79
$\lambda_{0}$	The basal encounter rate of a female lion whose activity center is located exactly at the centroid of a grid cell	0.003	0.001	0.001	0.005
ψ	Proportion of the true number of individuals in the data‐augmented population *M*	0.47	0.11	0.26	0.70
*N*_super_	The total number of lions in the larger state‐space *S*	116.84	27.33	64	169
$\psi_{\text{sex}}$	The proportion of lions that are male	0.32	0.12	0.10	0.57
*D*	Estimated density of lions/100 km^2^ > 1 year of age	5.87	1.37	3.32	8.60
N	Estimated abundance within survey area (358 km^2^)	21.04	4.92	12.06	30.97
$N_{1\sigma }$	Estimated abundance within larger area (474 km^2^) calculated by adding a buffer according to the weighted mean of the *σ* parameters	27.50	4.52	19	36

Note

Estimates presented above are from Model 1 $\left(\left[\beta_{\text{sex}},\theta \left(.\right)\left[.\right]\right.\right)$ and include posterior standard deviations and 95% highest posterior density intervals (HPD). Number of posterior samples used was 200,000. Maximum value of potential scale reduction factor = 1.01, Bayesian *p*‐value = .577. See Figure A3 for pairwise plots of parameters, A4 for posterior distributions, and Tables A1 and A2 for more detailed summaries from Models 1–4.

We ran the models in R (R Core Team, 2021), using the same priors as Broekhuis and Gopalaswamy (2016) and adapted the code provided by Elliot and Gopalaswamy (2017) to include the playback covariate. Each model was set to run for four chains, each with 51,000 iterations and an initial burn‐in of 1000 iterations. We assessed convergence using the Gelman‐Rubin diagnostic (Gelman & Rubin, 1992).

Our model choice was informed by a combination of three criteria (Elliot et al., 2020): Bayesian *p*‐value (Royle et al., 2009), the logarithm of the Marginal Likelihood using the Harmonic Mean estimator (MLHM, Dey, Delambady, & Gopalaswamy, 2019), and pairwise correlation plots between estimated parameters, where we were particularly concerned with correlations involving the density parameters. Input files and R code used to run the analyses are provided in Appendix 2.

### 2.5 Posterior mean abundance

A previous study in this landscape reported lion abundance and density estimates for a 250 km^2^ area that is completely encompassed within our larger survey area (358 km^2^) (Schuette et al., 2013). Although we did not have precise spatial information from that study, we were interested in discussing our results in the context of Schuette et al. (2013) and thus computed two abundance estimates: first, we calculated posterior mean abundance for our survey area using area (358 km^2^) multiplied by posterior mean density; second, we added a meaningful and conservative buffer around the sampled traps that was equivalent to the weighted mean of the estimated movement parameters ($\sigma_{\text{male}},\sigma_{\text{female}})$. For all iterations of the Markov Chain Monte Carlo (MCMC) outputs, we took the sum of all pixels within the buffer and computed posterior mean abundance. For both abundance estimates, we computed posterior standard deviation (PSD) and 95% highest posterior density intervals (HPD).

## 3 RESULTS

**FIGURE 1:**
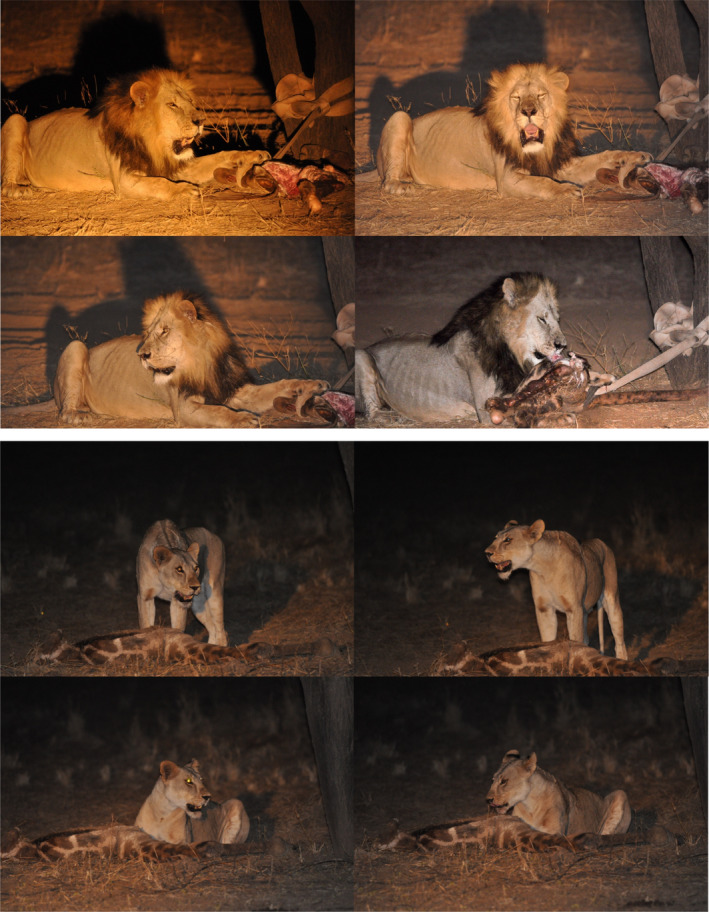
Two field protocols were deployed to find and identify individual lions within Shompole and Olkiramatian community areas, Kenya: a search encounter field protocol and a playback protocol. Our unstructured spatial capture‐recapture sampling design accounted for both protocols per pixel per sampling occasion (1 day) and resulted in 85 detections of 19 individuals (colored lines connecting detections represent spatial recaptures of individuals which have been jittered)

During the 89‐day survey, we drove 2701 km and conducted 14 playbacks in search of lions. This resulted in 84 detections of 19 individuals (6 males and 13 females) estimated to be >1 year old (Figure 1). We detected 17 individuals at more than one trap, with each individual being detected at an average at 3.1 traps. The vast majority of our 84 detections occurred during the search encounter protocol (81), while only 3 detections (two males and one female all on separate occasions) resulted from 3 different playbacks (see Figure A2 for example photographs). We detected 2 individuals once, 2 individuals twice, 1 individual 3 times, 10 individuals 5 times, 3 individuals 6 times, and 1 individual 7 times. Seven additional detections could not be identified to individual level and were discarded. Two thirds of the detections (*n* = 56) occurred during night‐time hours (19:00–06:00), while the rest occurred in the early hours of the morning (before 08:00) or late evening (after 18:40). Just over half of our fieldwork (51%) was conducted at night.

### 3.1 Model diagnostics

All models achieved convergence ($\hat{R}\leq 1.05$ for each parameter) with 50,000 iterations. Bayesian *p* value for all models was estimated between 0.5 and 0.58, which were well within the extremities (0.15–0.85, Table A1). The MLHM estimate (Dey, Delampady, & Gopalaswamy, 2019) was highest for Model 3 (Table A1), and visual inspection of the pairwise correlation plots between estimated parameters from the posterior MCMC draws showed minimal parameter redundancy across the models (Figure A3). Considering these pieces of evidence together, and since parameter estimates and levels of precision were very similar between all models (Table A2 and Figure A4), we report the posterior parameter estimates from the most informative model, Model 1.

### 3.2 Posterior summaries for lions >1 year old

**FIGURE 2:**
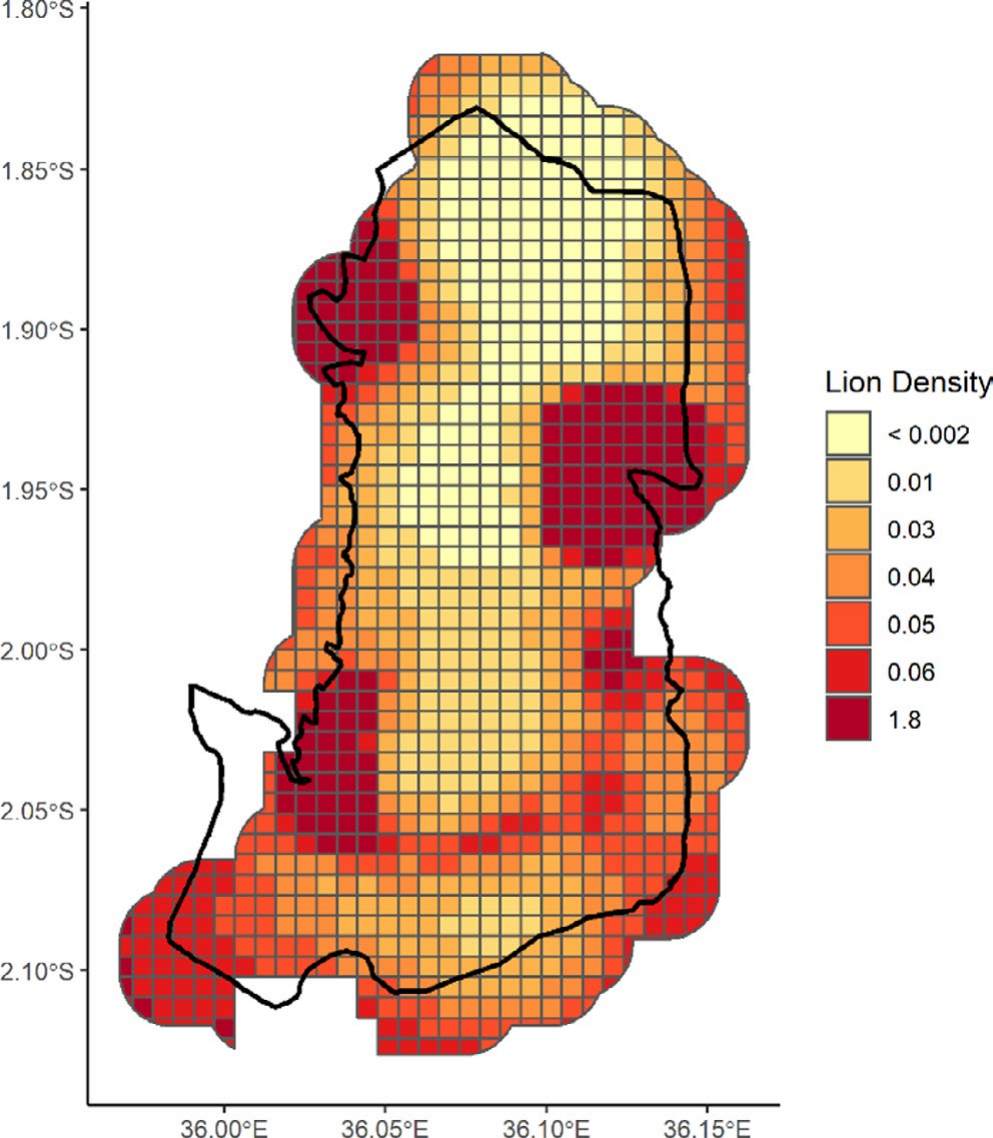
Pixel‐specific lion density expressed in units of individual lion activity centers per state‐space pixel (0.5 km^2^) in Shompole and Olkiramatian community areas in Kenya's South Rift Ecosystem. Estimated by Model 1, three hotspots of lion activity are revealed. The area for which pixel density is displayed was created based on the weighted mean of the posterior estimates for σ, and abundance was calculated both within the survey area (358 km^2^) and the larger buffer (474 km^2^)

Posterior mean lion density within our study area was estimated at 5.9 (PSD = 1.4, HPD = 3.3–8.6) individuals >1 year/100 km^2^. Lion abundance within the study area and larger buffer (474 km^2^) was estimated at 21 (PSD = 4.9, HPD = 12–31) and 27 (PSD = 4.5, HPD = 19–36), respectively. The sex ratio, as estimated by $\psi_{\text{sex}}$, was 2.1♀:1♂. Posterior summaries of all parameters estimated from Model 1, including the beta parameters for our field protocols, are displayed in Table 1. Detailed summaries of all models are provided in Table A2, and posterior density estimates for each 0.5 km^2^ pixel are displayed in Figure 2.

## 4 DISCUSSION

Our estimated lion density (5.9 individuals >1 year/100 km^2^) compares favorably to estimates using similarly robust methods and age classes within PAs (2.4/100 km^2^ in Queen Elizabeth NP, Uganda—Braczkowski et al., 2020; 6.7/100 km^2^ in Lake Nakuru NP, Kenya—Elliot et al., 2020). However, it is considerably lower than the 13.6 lions >1 year/100 km^2^ reported within an area encompassed by the current study (Schuette et al., 2013). However, we caution against comparing our estimates to those of Schuette et al. (2013) since they did not conduct a formal survey, but rather individually identified 34 lions within an arbitrarily defined area of 250 km^2^ over a period of 1 year and converted this to density. The prolonged timeframe is likely to have violated assumptions of demographic and geographic closure (Karanth & Nichols, 1998), and it is possible they detected lions that majorly resided outside their study area (see Figure A5 for collar data from around that period which supports this view). Furthermore, their approach did not allow them to eliminate detection bias. Our SCR framework overcomes these concerns by formally linking individuals and space and defines N within an explicit spatial region (the state‐space), allowing for direct estimates of density with a measure of precision while accounting for detection probability. However, we do note some numerical congruence between the 34 lions seen by Schuette et al. (2013) and our abundance estimate (27 lions) for the larger area.

The relatively high density of lions in the current study is noteworthy, given it is a landscape shared by wildlife, livestock, and people and is more than 100 km away from the nearest PA. That lions occur at this density may be partially explained by the tolerance of the local Maasai community for lions as reported by an attitudinal questionnaire survey showing that 88% of respondents expressed a desire for lion numbers to stay the same or increase (Western et al., 2019). This attitude was largely attributed to perceived benefits, such as increasing tourism revenue, despite livestock depredation by lions (Western et al., 2019). It is plausible that the broad range of community based conservation initiatives in this area have led to a perceived ownership of lions by the local community (Western, 2012) and enabled benefits to reach the community, thus fostering a landscape of coexistence (Western et al., 2019) where lions are not only tolerated, but actively conserved. This hypothesis finds support in the nearby Maasai Mara, where high densities of lions were reported in wildlife conservancies (Elliot & Gopalaswamy, 2017), and community member's attitudes to carnivores was generally positive, largely due to perceived benefits (Broekhuis et al., 2020). Finally, the relatively low livestock densities in the current survey area combined with the effective management of livestock and their seasonal movements by community grazing committees may help to facilitate coexistence (Russell et al., 2018; Tyrrell et al., 2017). Given that around 44% of existing lion range may lie outside formally PAs (Lindsey et al., 2018), our density estimates offer reason for optimism that lions may persist in such areas if the local community desires this.

In the current study, we set out to extend the field and analytical approaches provided by Elliot and Gopalaswamy (2017) to include playbacks, a commonly used field technique. This technique has commonly been deployed in a structured manner, usually referred to as “call‐in” or “playback” surveys to estimate the population size of African carnivores (Funston & Henschel, 2018; Ogutu & Dublin, 1998). This technique does not make use of individual identification and the estimates have low precision (Elliot & Gopalaswamy, 2017). We opportunistically used playbacks to attract lions to obtain individual identification photographs. We detected solitary lions during 3 of the 14 playbacks (two different males and one female). These were the only occasions that lions were observed during playbacks and we managed to successfully identify all individuals. While the presence of a carcass assisted in providing a distraction and anchor for the lions allowing us to photograph them, most detections during the search encounter protocol were at night when no carcass was present. Furthermore, other surveys using the same protocols have since been conducted without the use of baits, yielding a good number of detections (Elliot et al., 2022). Thus, a bait may be helpful, especially for recaptures but is not essential to obtaining detections during playbacks. Although the playbacks did not result in many detections, and consequently an insignificant effect (see $\beta_{\text{eff}2}$ in Table 1), we were able to successfully demonstrate how to combine this field protocol with the search encounter protocol and modeling framework of Elliot and Gopalaswamy (2017) while accounting for different detection rates arising from each field protocol. Our study also highlights the value of conducting large carnivore sampling during night‐time hours, a practice that is not always permitted. We foresee that this combined approach will have great utility in areas where lions are not habituated to vehicles and thus help to provide reliable lion density estimates for a wide variety of important source populations. SCR models generally assume that activity centers are independent of one another, which is unlikely to be the case with social animals such as lions. While simulation studies suggest that SCR models are largely robust to violations of this assumption (Bischof, Dupont, et al., 2020; López‐Bao et al., 2018; Russell et al., 2012), we note with interest a recently developed SCR model for group‐living species (Emmet et al., 2021). However, this model requires knowledge of which group each individual belongs to, which is frequently not known in field studies such as ours.

## 5 CONCLUSION AND RECOMMENDATIONS

The survey area we defined does not contain an isolated population with lions recently documented in Torosei and Musenge (~30–50 km east of the survey area, Western unpublished data), and in nearby Naimina Enkiyio Forest (~25 km west of the survey area, Broekhuis et al., 2018). Future local monitoring efforts should therefore aim to increase the sampling area, while also increasing the field effort, which would provide valuable information and likely have the added advantage of increasing the relatively low precision of our estimates, since more recaptures are likely to be recorded. In addition, a wet season sampling session is recommended to gain an understanding of potential seasonal differences also noting that this is a multiple‐use landscape. At a landscape level, it is possible that lions are distributed across southern Kenya from the Maasai Mara to the Tsavo ecosystem (Broekhuis et al., in review). Population monitoring across this area is recommended and could be achieved using the methods presented in this paper, perhaps within a combined SCR approach (Gopalaswamy, Royle, Delampady, et al., 2012) that makes use of high‐quality camera traps, unstructured sampling to collect noninvasive DNA via fecal samples (e.g., Bischof, Milleret, et al., 2020), or by using sign‐based occupancy surveys (e.g., Karanth et al., 2011).

## CONFLICT OF INTEREST

The authors declare no conflict of interest.

## AUTHOR CONTRIBUTIONS

**Guy Western:** Conceptualization (equal); Data curation (equal); Formal analysis (equal); Funding acquisition (equal); Investigation (equal); Methodology (equal); Project administration (equal); Supervision (equal); Validation (equal); Visualization (equal); Writing – original draft (equal); Writing – review & editing (equal). **Nicholas B. Elliot:** Conceptualization (equal); Data curation (equal); Formal analysis (equal); Funding acquisition (equal); Investigation (equal); Methodology (equal); Project administration (equal); Software (equal); Supervision (equal); Validation (equal); Visualization (equal); Writing – original draft (equal); Writing – review & editing (equal). **Steiner L. Sompeta:** Conceptualization (supporting); Formal analysis (supporting); Validation (supporting); Writing – review & editing (supporting). **Femke Broekhuis:** Conceptualization (equal); Data curation (equal); Funding acquisition (equal); Investigation (equal); Methodology (equal); Project administration (equal); Software (equal); Validation (equal); Writing – review & editing (equal). **Shadrack Ngene:** Conceptualization (equal); Funding acquisition (equal); Project administration (equal); Writing – review & editing (equal). **Arjun M. Gopalaswamy:** Conceptualization (equal); Formal analysis (equal); Funding acquisition (equal); Investigation (equal); Methodology (equal); Project administration (equal); Software (equal); Supervision (equal); Validation (equal); Writing – review & editing (equal).

## Supporting information

Appendix S1

Appendix S2

## Data Availability

All input files and R scripts to reproduce our analysis are available in Appendix 2.
