# Supplementary material for: Lions in a coexistence landscape: Repurposing a traditional field technique to monitor an elusive carnivore
Source: Ecol Evol. 2022 Mar 1;12(3):e8662. doi: 10.1002/ece3.8662 (PMC8888262; doi:10.1002/ece3.8662)
Supplement: Supplementary file 1 — Appendix S1 [file ECE3-12-e8662-s002.docx]

Appendix 1

Figure A1. The lion state-space (~2,358 km^2^) as defined by a 15 km buffer around the survey area (358 km^2^) in Shompole and Olkiramatian community areas in Kenya’s South Rift Ecosystem. Agricultural areas and large water bodies were masked out as unsuitable lion habitat prior to analysis.


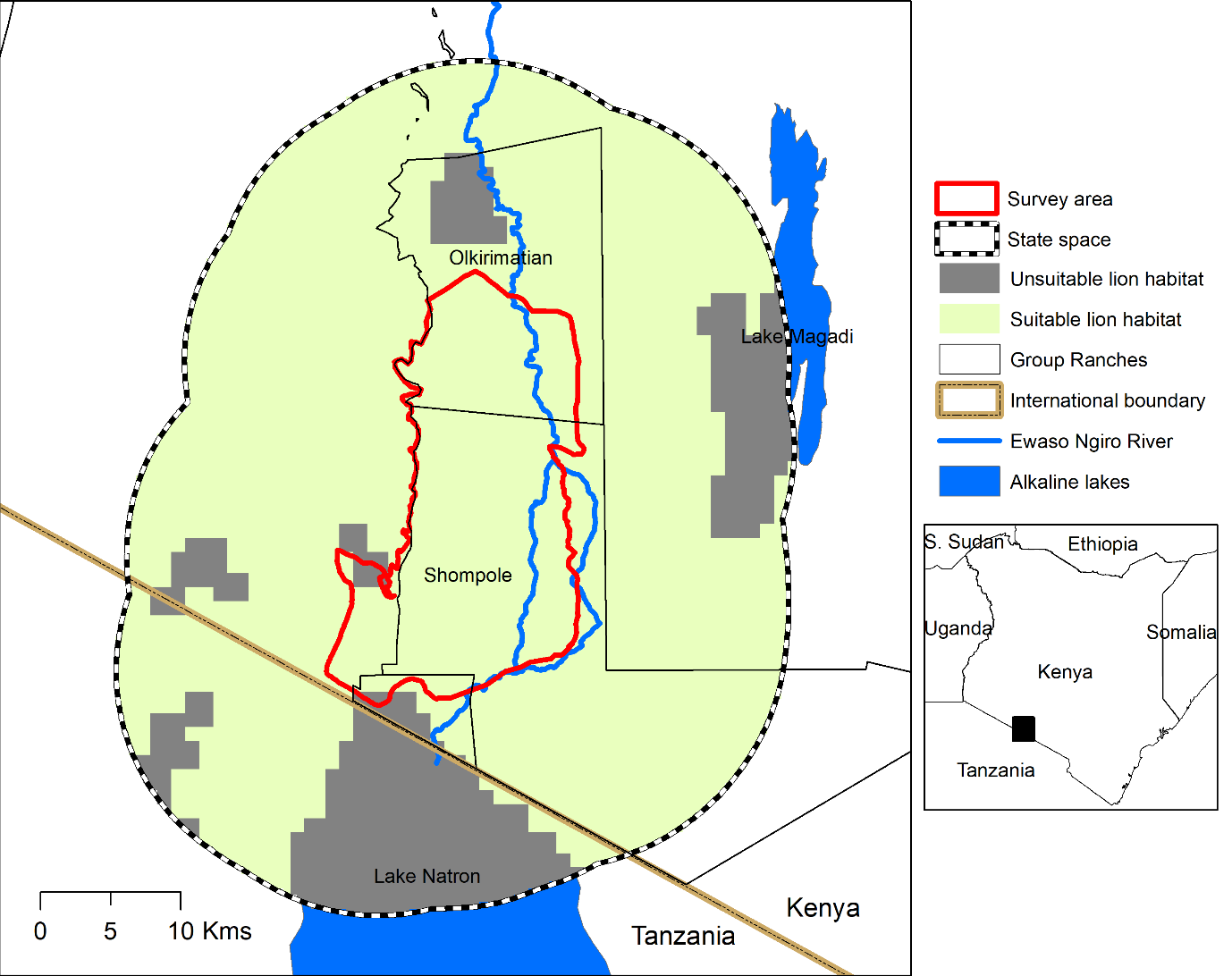


**
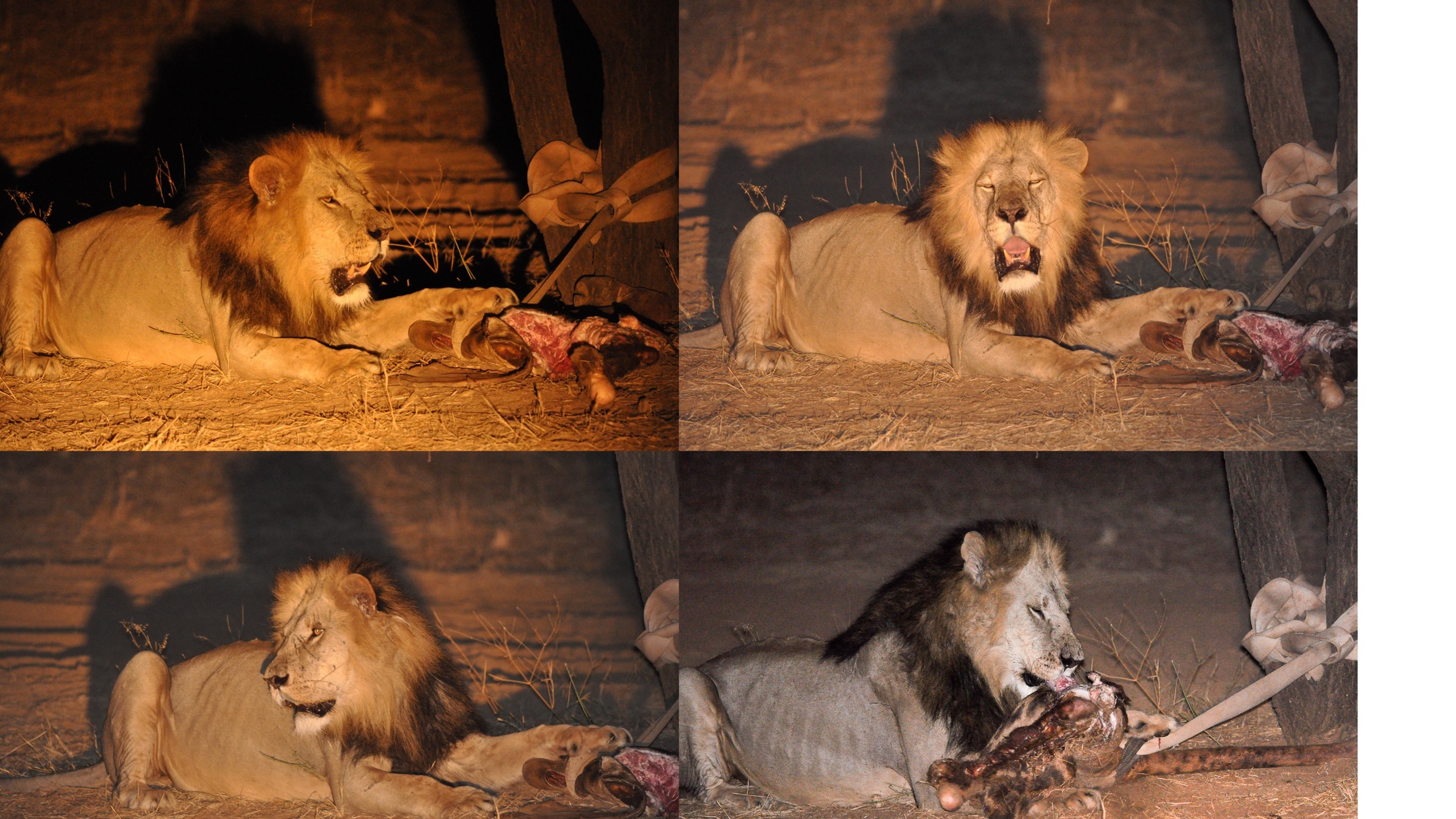
**Figure A2i. A selection of photographs taken of M01 during a playback conducted on 13/10/2018 that were used for individual identification. This male lion was attracted to distress calls broadcast from a speaker during the playback field protocol. A section of an opportunistically collected giraffe carcass had been tied to a tree, providing a distraction for the lion, and allowing the field team to obtain individual identification photographs.

**
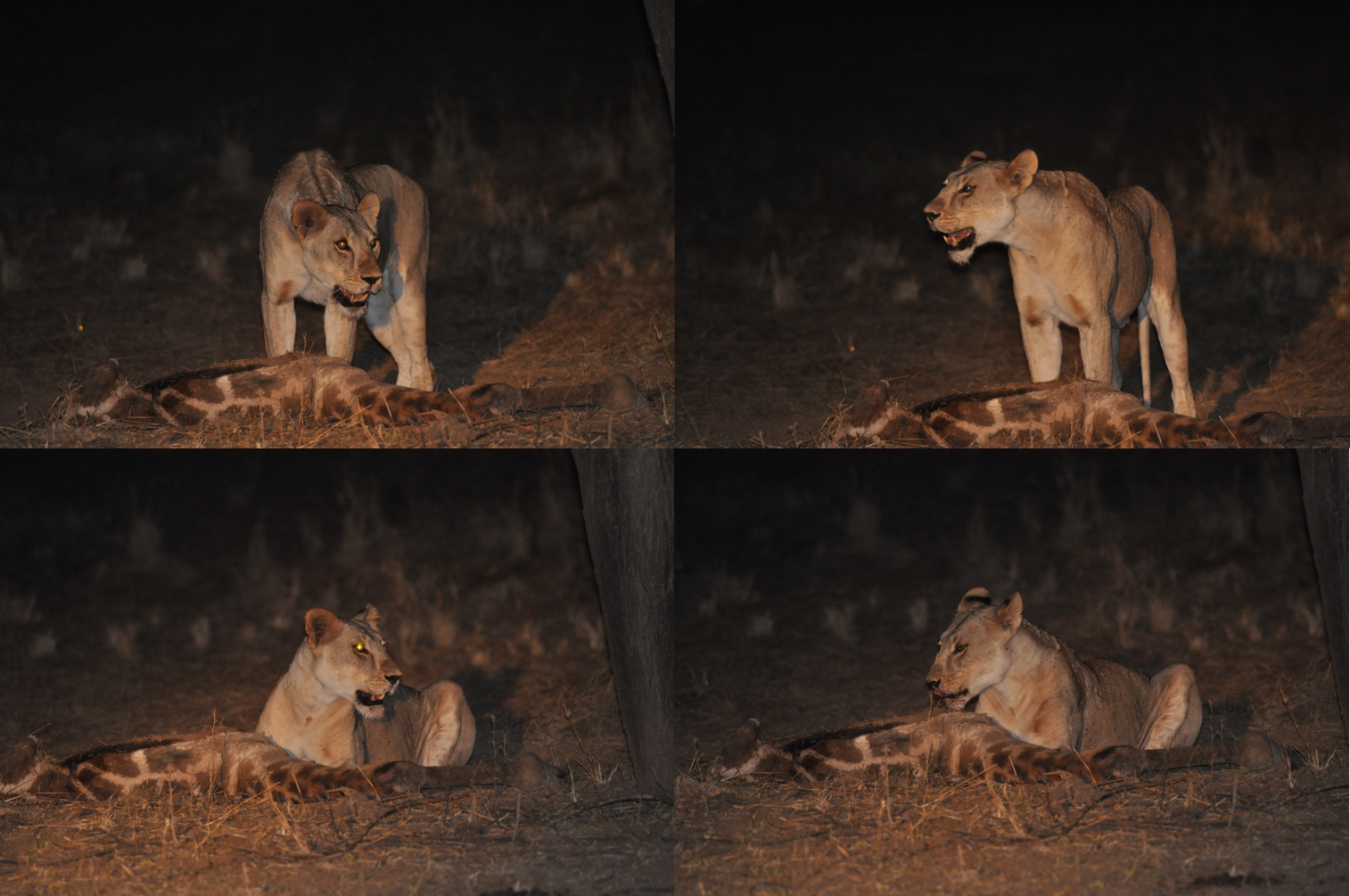
**Figure A2ii. A selection of photographs taken of F03 during a playback conducted on 16/10/2018 that were used for individual identification.

Figure A3 i-iv. Pairwise plots between estimated parameters from the posterior MCMC draws. These were used to visually assess covariance and parameter redundancy (or identifiability) issues as a result of model overfitting relative to sample size. Since density (*D.adj*) was of primary interest to our study, we were particularly concerned to assess whether there were correlations between these parameters and any other parameter.

1. Model 1: $N\left( . \right),\lambda_{0}\left( sex+effort+effort_{2} \right), \sigma(sex)$


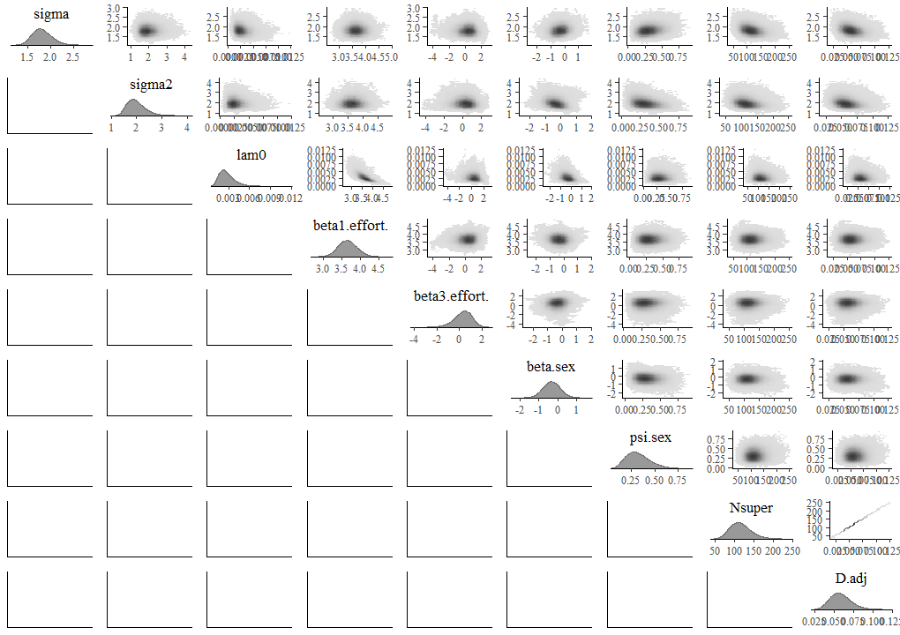


1. Model 2: $N\left( . \right),\lambda_{0}\left( effort+effort_{2} \right), \sigma(sex)$


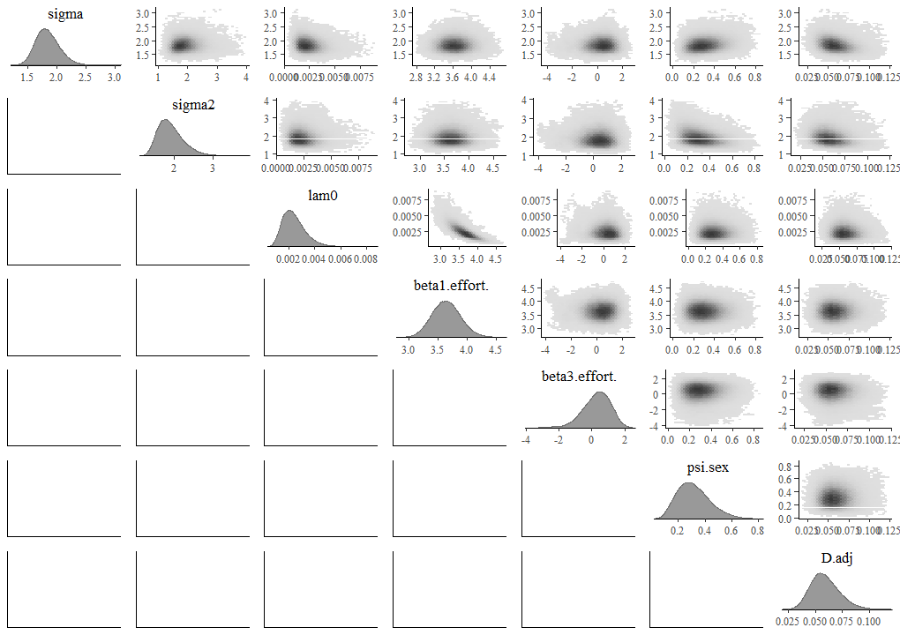


1. Model 3: $N\left( . \right),\lambda_{0}\left( effort+effort_{2} \right), \sigma\left( . \right)$


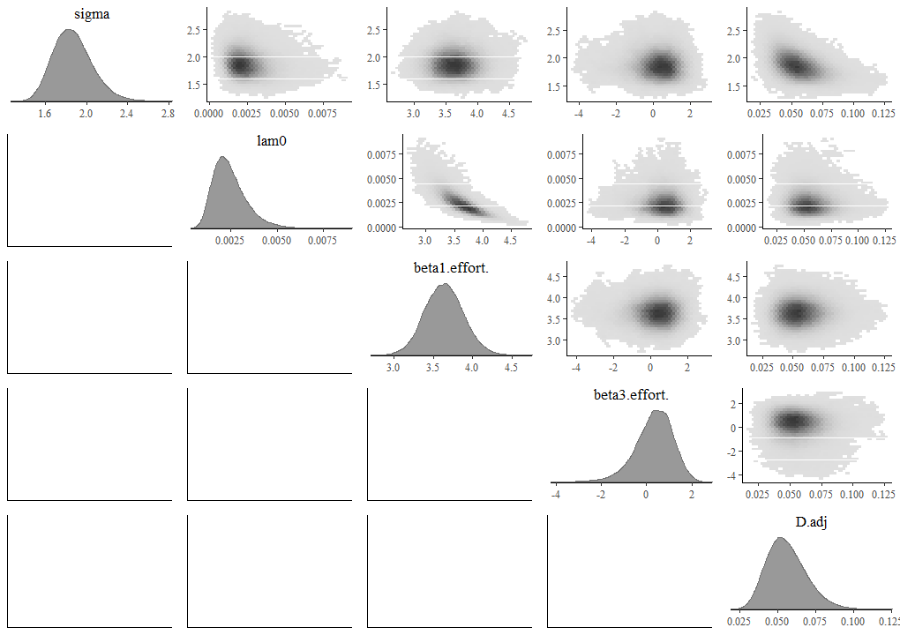


1. Model 4: $N\left( . \right),\lambda_{0}\left( sex+effort+effort_{2} \right), \sigma(.)$


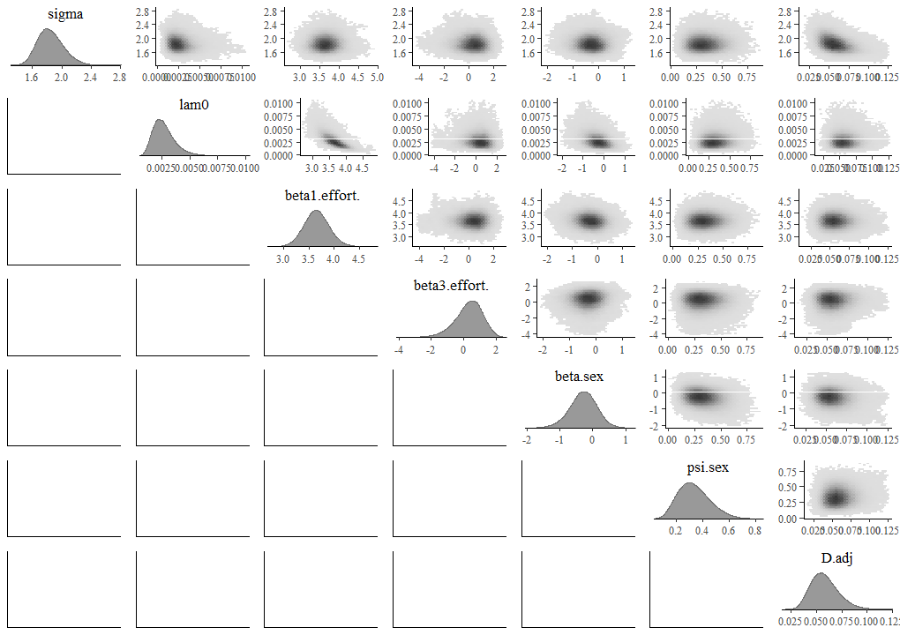


Figure A4 i-iv. Distribution of Bayesian posterior density estimates from Models 1 – 4 for lions in Shompole and Olkiramatian community areas, Kenya. The blue dotted line denotes the mean, the red dotted line denotes the median, the green dotted line denotes the mode, and the black line denotes the 95% lower and upper highest posterior density intervals. We set four chains to run for each model, with 51,000 iterations. We discarded outcomes from the first 1,000 iterations as burn-in. The plots were created using the ‘geom_density’ function in the ggplot2 library (Wickham 2016) and display a smoother kernel density estimate instead of count values on the y-axis.

1. Model 1: $N\left( . \right),\lambda_{0}\left( sex+effort+effort_{2} \right), \sigma(sex)$


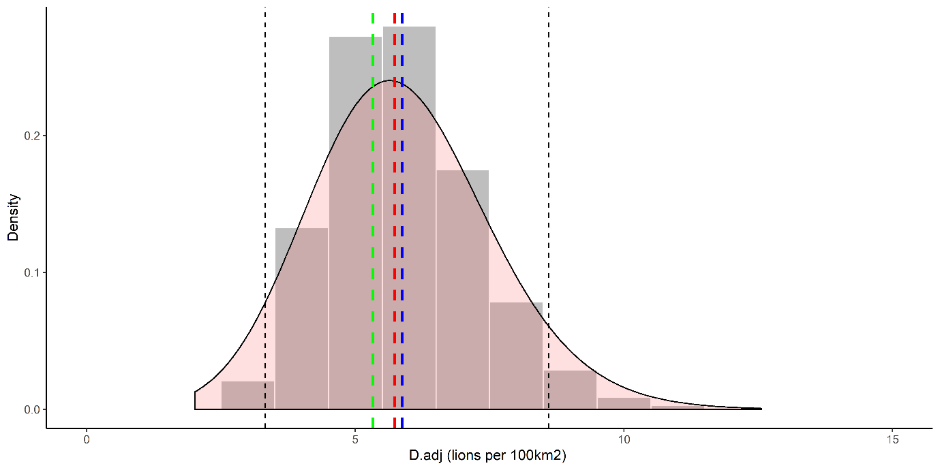


1. Model 2: $N\left( . \right),\lambda_{0}\left( effort+effort_{2} \right), \sigma(sex)$


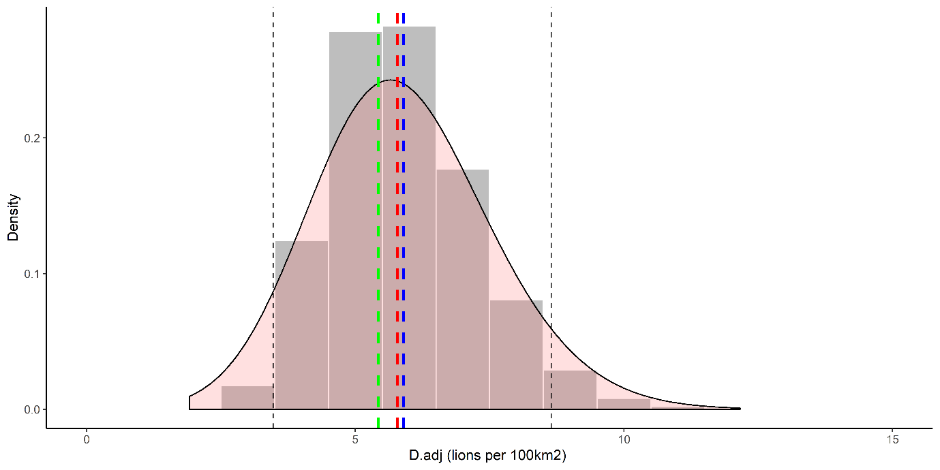


1. Model 3: $N\left( . \right),\lambda_{0}\left( effort+effort_{2} \right), \sigma\left( . \right)$


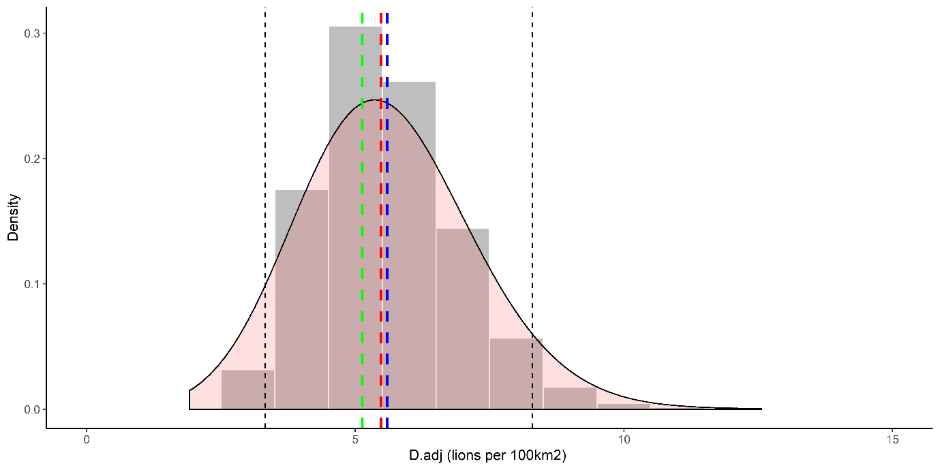


1. Model 4: $N\left( . \right),\lambda_{0}\left( sex+effort+effort_{2} \right), \sigma(.)$


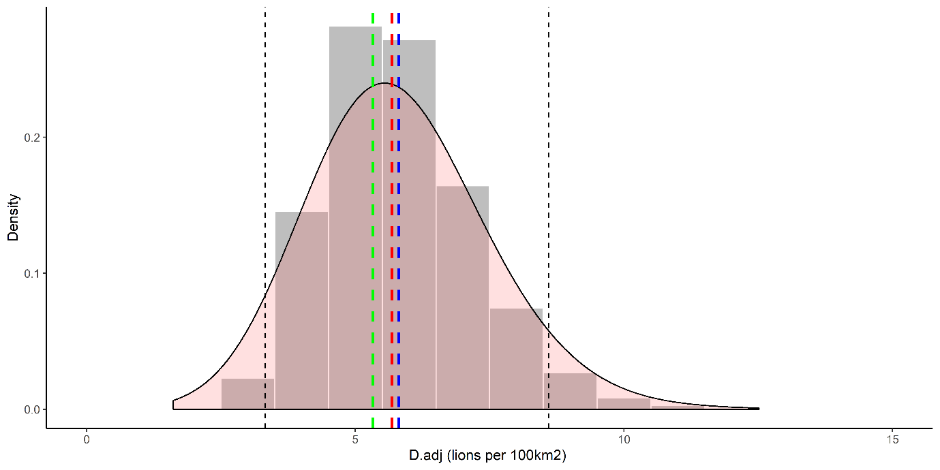


Figure A5. Maps showing the surveyed area together with GPS telemetry data from three lions that were collared between 2011-2013. This is a similar period to that of Schuette et al. (2013) who, in 2010, individually identified 34 lions within an arbitrarily defined area of 250km^2^ over a period of one year and converted this to a density of 13.6 lions > 1 year old / 100km^2^. These maps show three lions using the survey area to varying degrees and illustrate why randomly defining a survey area and converting to density based on whole count data is not meaningful. Our SCR framework overcomes these concerns by formally linking individuals and space, and defines N within an explicit spatial region (the state-space), allowing for direct estimates of density with a measure of precision while accounting for detection probability.


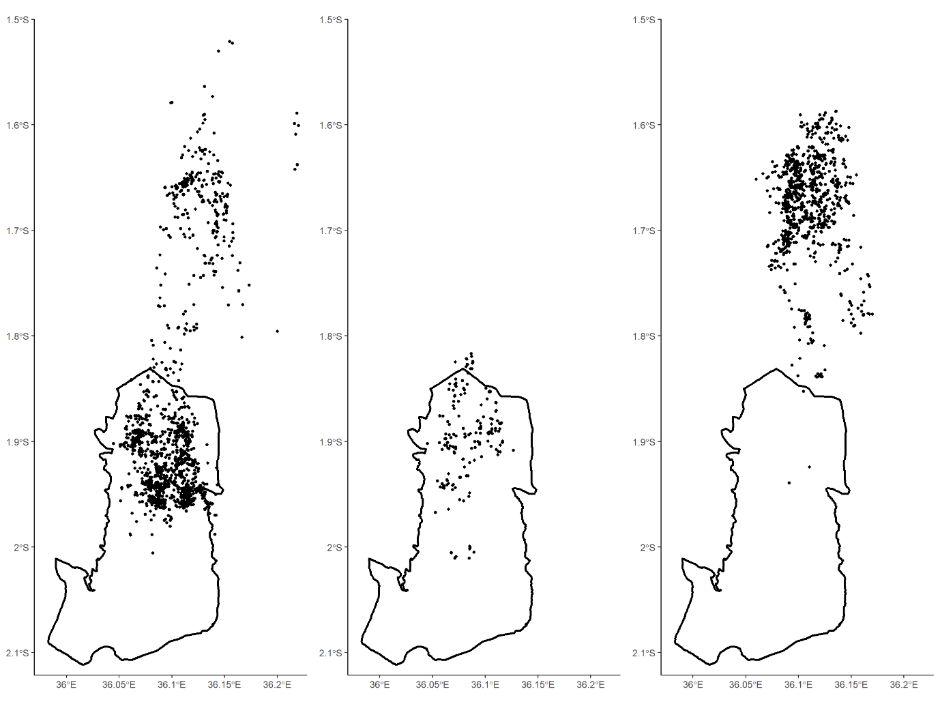


Table A1. Model specification and diagnostics for Models 1-4.

|  | **M1** | **M2** | **M3** | **M4** |
| --- | --- | --- | --- | --- |
| **Parameter** | **Setting** | **Setting** | **Setting** | **Setting** |
| Msex | 1 | 0 | 0 | 1 |
| Msexsigma | 1 | 1 | 0 | 0 |
| Msigma | 1 | 1 | 1 | 1 |
| Theta | 1 | 1 | 1 | 1 |
| Nz | 231 | 231 | 231 | 231 |
| Iterations | 51,000 | 51,000 | 51,000 | 51,000 |
| Burn | 1,000 | 1,000 | 1,000 | 1,000 |
| Post hoc burn | 0 | 0 | 0 | 0 |
| Max. scale reduction factor | 1.01 | 1.01 | 1.01 | 1.01 |
| Bayes P-value | 0.577 | 0.545 | 0.503 | 0.574 |
| Marginal likelihood | -66067 | -61910 | -56341 | -61036 |

Table A2 i-iv. Posterior estimates of parameters for Models 1-4. Model 1 was selected for reporting due to (a) Bayesian *p* value lying within the extremities (0.15-0.85), (b) minimal pairwise correlations and (c) all models produced very similar estimates, hence we report the model with most information. See manuscript for definition of parameters, Appendix 2 for R scripts and input files and Figure A4 for pairwise plots.

1. Model 1: $N\left( . \right),\lambda_{0}\left( sex+effort+effort_{2} \right), \sigma(sex)$

|  | Mean | Mode | Median | Post. SD | Monte Carlo SE | Lower 95% HPDI | Upper 95% HPDI |
| --- | --- | --- | --- | --- | --- | --- | --- |
| $\sigma_{F}$  $\sigma_{M}$ | 1.82 | 1.80 | 1.80 | 0.21 | 0.00 | 1.42 | 2.23 |
|  | 2.00 | 2.26 | 1.95 | 0.37 | 0.01 | 1.36 | 2.75 |
| $\lambda_{0}$ | 0.003 | 0.004 | 0.003 | 0.001 | 0.000 | 0.001 | 0.005 |
| $\beta_{eff}$ | 3.65 | 3.68 | 3.64 | 0.25 | 0.01 | 3.15 | 4.14 |
| $\beta_{eff2}$ | 0.28 | 2.46 | 0.35 | 0.83 | 0.03 | -1.46 | 1.79 |
| $\beta_{sex}$ | -0.33 | -0.73 | -0.33 | 0.45 | 0.01 | -1.21 | 0.55 |
| $\psi_{sex}$  $\psi$ | 0.47 | 0.41 | 0.46 | 0.11 | 0.00 | 0.26 | 0.70 |
| $N_{super}$ | 0.32 | 0.39 | 0.31 | 0.12 | 0.00 | 0.10 | 0.57 |
|  | 116.84 | 106.00 | 114.00 | 27.33 | 0.51 | 64.00 | 169.00 |
| $D$ | 5.87 | 5.33 | 5.73 | 1.37 | 0.03 | 3.32 | 8.60 |

1. Model 2: $N\left( . \right),\lambda_{0}\left( effort+effort_{2} \right), \sigma(sex)$

|  | Mean | Mode | Median | Post. SD | Monte Carlo SE | Lower 95% HPDI | Upper 95% HPDI |
| --- | --- | --- | --- | --- | --- | --- | --- |
| $\sigma_{F}$  $\sigma_{M}$ | 1.85 | 2.08 | 1.83 | 0.21 | 0.00 | 1.47 | 2.26 |
|  | 1.91 | 1.23 | 1.86 | 0.34 | 0.01 | 1.33 | 2.60 |
| $\lambda_{0}$ | 0.002 | 0.002 | 0.002 | 0.001 | 0.000 | 0.001 | 0.004 |
| $\beta_{eff}$ | 3.64 | 3.77 | 3.63 | 0.24 | 0.01 | 3.16 | 4.11 |
| $\beta_{eff2}$ | 0.28 | 2.26 | 0.36 | 0.86 | 0.03 | -1.40 | 1.90 |
| $\beta_{sex}$ | NA | NA | NA | NA | NA | NA | NA |
| $\psi_{sex}$  $\psi$ | 0.47 | 0.53 | 0.46 | 0.11 | 0.00 | 0.27 | 0.69 |
| $N_{super}$ | 0.31 | 0.31 | 0.30 | 0.12 | 0.00 | 0.10 | 0.55 |
|  | 117.23 | 108.00 | 115.00 | 26.72 | 0.51 | 66.00 | 169.00 |
| $D$ | 5.89 | 5.43 | 5.78 | 1.34 | 0.03 | 3.47 | 8.65 |

1. Model 3: $N\left( . \right),\lambda_{0}\left( effort+effort_{2} \right), \sigma\left( . \right)$

|  | Mean | Mode | Median | Post. SD | Monte Carlo SE | Lower 95% HPDI | Upper 95% HPDI |
| --- | --- | --- | --- | --- | --- | --- | --- |
| $\sigma_{F}$  $\sigma_{M}$ | 1.87 | 1.85 | 1.85 | 0.19 | 0.005 | 1.51 | 2.24 |
|  |  |  |  |  |  |  |  |
| $\lambda_{0}$ | 0.002 | 0.002 | 0.002 | 0.001 | 0.000 | 0.001 | 0.004 |
| $\beta_{eff}$ | 3.64 | 4.22 | 3.63 | 0.25 | 0.01 | 3.17 | 4.13 |
| $\beta_{eff2}$ | 0.31 | 1.94 | 0.39 | 0.86 | 0.03 | -1.38 | 1.93 |
| $\beta_{sex}$ | NA | NA | NA | NA | NA | NA | NA |
| $\psi_{sex}$  $\psi$ | 0.45 | 0.41 | 0.44 | 0.11 | 0.00 | 0.25 | 0.66 |
| $N_{super}$ | NA | NA | NA | NA | NA | NA | NA |
|  | 111.22 | 102.00 | 109.00 | 26.07 | 0.49 | 62.00 | 161.00 |
| $D$ | 5.59 | 5.13 | 5.48 | 1.31 | 0.02 | 3.32 | 8.29 |

1. Model 4: $N\left( . \right),\lambda_{0}\left( sex+effort+effort_{2} \right), \sigma(.)$

|  | Mean | Mode | Median | Post. SD | Monte Carlo SE | Lower 95% HPDI | Upper 95% HPDI |
| --- | --- | --- | --- | --- | --- | --- | --- |
| $\sigma_{F}$  $\sigma_{M}$ | 1.84 | 1.97 | 1.82 | 0.18 | 0.01 | 1.51 | 2.19 |
|  |  |  |  |  |  |  |  |
| $\lambda_{0}$ | 0.003 | 0.003 | 0.003 | 0.001 | 0.000 | 0.001 | 0.005 |
| $\beta_{eff}$ | 3.65 | 3.67 | 3.65 | 0.24 | 0.01 | 3.18 | 4.13 |
| $\beta_{eff2}$ | 0.32 | 2.36 | 0.39 | 0.80 | 0.03 | -1.30 | 1.83 |
| $\beta_{sex}$ | -0.29 | -1.10 | -0.28 | 0.42 | 0.01 | -1.14 | 0.52 |
| $\psi_{sex}$  $\psi$ | 0.47 | 0.81 | 0.46 | 0.11 | 0.00 | 0.26 | 0.70 |
| $N_{super}$ | 0.33 | 0.43 | 0.32 | 0.12 | 0.00 | 0.12 | 0.57 |
|  | 116.60 | 108.00 | 114.00 | 27.88 | 0.76 | 65.00 | 170.00 |
| $D$ | 5.86 | 5.43 | 5.73 | 1.40 | 0.04 | 3.32 | 8.60 |

**References**

Schuette, P., S. Creel, and D. Christianson. 2013. Coexistence of African lions, livestock, and people in a landscape with variable human land use and seasonal movements. Biological Conservation **157**:148–154.

Wickham, H. 2016. ggplot2: Elegant Graphics for Data Analysis. Springer-Verlag New York.
