# Supplementary material for: Lions in a coexistence landscape: Repurposing a traditional field technique to monitor an elusive carnivore
Source: Ecol Evol. 2022 Mar 1;12(3):e8662. doi: 10.1002/ece3.8662 (PMC8888262; doi:10.1002/ece3.8662)
Supplement: Supplementary file 2 — Appendix S2 [file ECE3-12-e8662-s001.zip › ece38662-sup-0002-Appendix S2.rtf]

INSTRUCTIONS TO RUN THE R CODES AND REPRODUCE THE ANALALYSIS FROM WESTERN ET AL. SECR ANALYSIS FOR LIONS

Note: To perform this analysis, we recommend the use of a “supercomputer” or computer with a large RAM, and preferably a LINUX system. If running the current formulation, users can expect a model run-time of ~24 hours. 

FILES

In this zipped file, apart from this Instructions file, there are 10 other files. These are classified as: 

1.	The analysis functions (4 files)
a)	M1.R - This is the wrapper function and only script that needs to be run by the user and calls the below R functions. Here the user defines the input files and specifies the model to be run. In this example, sex effects are turned on and theta is fixed at 1. 
b)	SCRi.fn.par1-lionVer1003.R - This is the analysis engine, and is scripted to identify the OS and parallelise the analysis. 
c)	e2dist.R - A utility function necessary for certain computations.
d)	scrData.R - A data formatting function

2.	Data input files (5 files needed to run the SECR analysis)
a)	Habitat.csv - This is the statespace
b)	Traps.csv - This is the pixel (trap) operations matrix
c)	CH.csv - Lion capture-histories on pixels
d)	Sex.csv - Sex identification file of lion individuals
e)	Effort.csv - Search encounter effort investment file
f)	Effort2.csv - Playback effort investment file 

RUNNING INSTRUCTIONS

1.	Store the above 10 files in a directory and in R, change directory to this directory. 
2.	The user will only have to run the R script (M1.R). This script calls all the other functions and data files and runs the analysis. Before running this, the user must change the reference directory to store output files to his/her own directory in the code. 
3.	Once the run is complete, results are stored in various directories. 
